# Clinical importance of B7-H3 expression in human pancreatic cancer

**DOI:** 10.1038/sj.bjc.6605375

**Published:** 2009-10-20

**Authors:** I Yamato, M Sho, T Nomi, T Akahori, K Shimada, K Hotta, H Kanehiro, N Konishi, H Yagita, Y Nakajima

**Affiliations:** 1 Department of Surgery, Nara Medical University, Nara, Japan; 2 Department of Pathology, Nara Medical University, Nara, Japan; 3 Renal and Genitourinary Surgery, Hokkaido University Graduate School of Medicine, Sapporo, Japan; 4 Department of Immunology, Juntendo University School of Medicine, Tokyo, Japan

**Keywords:** B7-H3, pancreas, immunotherapy, T-cell, antibody

## Abstract

**Background:**

B7-H3 is a new member of the B7 ligand family and regulates T-cell responses in various conditions. However, the role of B7-H3 in tumour immunity is largely unknown. The purpose of this study was to evaluate the clinical significance of B7-H3 expression in human pancreatic cancer and the therapeutic potential for cancer immunotherapy.

**Methods:**

We investigated B7-H3 expression in 59 patients with pancreatic cancer by immunohistochemistry and real-time PCR. Furthermore, we examined the anti-tumour effect of B7-H3-blocking monoclonal antibody *in vivo* in a murine pancreatic cancer model.

**Results:**

Tumour-related B7-H3 expression was abundant in most human pancreatic cancer tissues and was significantly higher compared with that in non-cancer tissue or normal pancreas. Moreover, its expression was significantly more intense in cases with lymph node metastasis and advanced pathological stage. B7-H3 blockade promoted CD8^+^ T-cell infiltration into the tumour and induced a substantial anti-tumour effect on murine pancreatic cancer. In addition, the combination of gemcitabine with B7-H3 blockade showed a synergistic anti-tumour effect without overt toxicity.

**Conclusion:**

Our data show for the first time that B7-H3 may have a critical role in pancreatic cancer and provide the rationale for developing a novel cancer immunotherapy against this fatal disease.

Pancreatic cancer is one of most aggressive and intractable human malignant tumours and a leading cause of cancer-related deaths worldwide (Li *et al*, 2004; Laheru and Jaffee, 2005). Owing to its extremely high malignant potential, it is usually diagnosed at an advanced stage and often recurs even after curative surgery (Traverso, 2006). Although recent significant advances in cancer therapy, including the introduction of new chemotherapeutic agents, have made a significant impact on the overall survival of pancreatic cancer patients, complete cure is very rare and the incidence rates are almost equal to mortality rates (Burris *et al*, 1997; Li *et al*, 2004; Laheru and Jaffee, 2005). Therefore, novel approaches against pancreatic cancer need to be developed and established to improve patient prognosis.

B7-H3 (CD276) is a new member of the B7 family and shares 20–27% identical amino acids with other members (Chapoval *et al*, 2001). B7-H3 is not expressed on quiescent lymphocytes and can be induced in activated dendritic cells, monocytes, T cells, and in some tumour cell lines (Chapoval *et al*, 2001; Sun *et al*, 2002; Steinberger *et al*, 2004; Zhang *et al*, 2005). B7-H3 is thought to serve as an accessory co-regulator of T-cell responses after initial antigen priming. At present, there is no consensus with regard to the physiological and pathological roles of B7-H3, as both immunological stimulatory and inhibitory functions have been described (Chapoval *et al*, 2001; Sun *et al*, 2002; Suh *et al*, 2003; Castriconi *et al*, 2004; Prasad *et al*, 2004; Steinberger *et al*, 2004; Wang *et al*, 2005; Zhang *et al*, 2005). It was originally identified as a co-stimulatory molecule that promotes T-cell proliferation and IFN-*γ* production (Chapoval *et al*, 2001). The experimental evidence that acute and chronic cardiac allograft rejection can be reduced in B7-H3 knockout mice further supports a stimulatory role for B7-H3 on T cells (Wang *et al*, 2005). By contrast, B7-H3 has been shown to impair type 1 T-helper cell responses and inhibit cytokine production (Suh *et al*, 2003). *In vivo* antibody-mediated blockade of B7-H3 in mice has been reported to enhance T-cell activation and to lead to more severe forms of experimental autoimmune encephalitis (Suh *et al*, 2003; Prasad *et al*, 2004). In addition, B7-H3 has been implicated to confer protection from natural killer (NK) cell-mediated cytolysis (Castriconi *et al*, 2004). Therefore, it is likely that B7-H3 exerts dual functions under certain circumstances.

In tumour immunity, the precise role of B7-H3 also remains unclear. B7-H3 expression has been identified in several tumour cell lines and in actual human tumour specimens, including gastric cancer, non-small-cell lung cancer, and prostate cancer (Sun *et al*, 2006; Wu *et al*, 2006; Roth *et al*, 2007; Zang *et al*, 2007). Several murine studies have shown that introduction of B7-H3 in tumour cells and tissues by various methods activates tumour-specific immunocompetent cells and promotes anti-tumour response, thereby leading to rapid tumour regression, reduction of metastasis, and prolongation of animal survival (Sun *et al*, 2003; Luo *et al*, 2004, 2006; Lupu *et al*, 2006, 2007). In addition, high intra-tumour B7-H3 expression was shown to correlate with better prognosis in gastric cancer (Wu *et al*, 2006). These data suggested that tumour-associated B7-H3 expression might promote immune response as a positive regulator. By sharp contrast, the negative role of B7-H3 expression has been reported in lung and prostate cancers (Sun *et al*, 2006; Roth *et al*, 2007; Zang *et al*, 2007). In non-small-cell lung cancer, B7-H3 expression was inversely correlated with the number of tumour-infiltrating lymphocytes, and a high B7-H3 expression was more common in patients with lymph node metastasis (Sun *et al*, 2006). Furthermore, a strong intensity for B7-H3 was significantly correlated with disease spread at the time of surgery, as well as with increased risk of recurrence and poor prognosis in prostate cancer (Roth *et al*, 2007; Zang *et al*, 2007). In addition, 4Ig-B7-H3 (B7-H3 protein with four-Ig-like domains) was identified in advanced stage neuroblastoma and has been shown to exert a protective effect on neuroblastoma cells from NK-mediated lysis (Castriconi *et al*, 2004). Thus, experimental and clinical data are conflicting and the precise role of B7-H3 in tumour immunity has not been fully elucidated yet.

In this study, we investigated the clinical significance of B7-H3 expression in human pancreatic cancer and the therapeutic efficacy of targeting the B7-H3 pathway towards future clinical application for the treatment of pancreatic cancer.

## Materials and methods

### Patients

We examined 59 patients with pancreatic cancer who underwent surgery at the Department of Surgery, Nara Medical University, between 1996 and 2004 (Table 1). The median age of the patients was 66 years, with a range of 42–80 years. Tissues, both cancerous and non-cancerous, were obtained from resected specimens and were then rapidly frozen at −80°C for storage until use. The remainder of each specimen was fixed in 10% phosphate-buffered formalin and embedded in paraffin. Pancreatic cancers were classified according to the TNM staging system. Post-operative pathological examinations have indicated metastasis in distant lymph nodes (M1, Stage IV) in seven patients. Primary tumour tissues from these patients were included for histological analysis in this study. Follow-up was until death or December 2007. The median follow-up for all patients was 14 months, with a range of 3–157 months. Most of the patients received systemic adjuvant chemotherapy after surgery. Normal pancreatic tissues were obtained from surgical specimens other than pancreatic cancer. Written informed consent was obtained from all patients before treatment, according to our institutional guidelines.

### Immunohistochemistry

Formalin-fixed, paraffin-embedded tissues of primary tumour were cut into 5-*μ*m sections, deparaffinised, and rehydrated in a graded series of ethanol. Antigen retrieval was carried out by heating tissue sections using a Target Retrieval Solution, pH 9.0 (DAKO, Tokyo, Japan). To block endogenous peroxidase, sections were immersed in 3% solution of hydrogen peroxide in absolute methanol for 10 min at room temperature and washed thrice in fresh PBS, each of 5 min duration. Purified goat anti-human B7-H3 antibody (100 *μ*g ml^−1^; R&D Systems, Minneapolis, MN, USA) diluted 1 : 10 with Antibody Diluent (DAKO) was added and incubated overnight at 4°C. Sections were washed thrice in PBS, each of 5 min duration, and then Histofine Simple Stain MAX PO (G) (NICHIREI, Tokyo, Japan) was added and incubated at room temperature for 30 min. After washing thrice, the Histofine DAB substrate kit (NICHIREI) was added and incubated at room temperature for 20 min. Sections were rinsed thrice in distilled water, counterstained with haematoxylin, dehydrated in ethanol, cleared in xylene, and coverslipped. For the staining of mouse CD8^+^ T cells, purified rabbit anti-CD8 antibody (Abcam, Tokyo, Japan) diluted 1 : 250 with Antibody Diluent (DAKO) was used and incubated overnight at 4°C. Sections were washed thrice in PBS, followed by incubation with the EnVision detection system (DAKO), according to the instructions of the manufacturer.

### Evaluation of immunostaining

Immunohistochemistry for B7-H3 was evaluated by authorised pathologists who had no knowledge of the patients’ clinical status and outcome. Each sample was thoroughly evaluated at a magnification of × 100. To count CD8^+^ T cells in murine pancreatic cancer tissue, three randomly selected areas were counted at × 200 magnification, and the average count was calculated.

### Extraction of total RNAs and real-time reverse-transcriptase PCR analysis

Total RNA was isolated using RNAspin Mini (GE Healthcare, Tokyo, Japan) and the first-strand cDNA was synthesised from 1 *μ*g RNA using a High Capacity cDNA Reverse Transcription Kit (Applied Biosystems, Foster City, CA, USA), according to the instructions of the manufacturer. Real-time quantitative PCR analysis was carried out using an ABI Prism 7700 sequence detector system (Applied Biosystems). All primer/probe sets were purchased from Applied Biosystems. PCR was carried out using the TaqMan Universal PCR Master Mix (Applied Biosystems) using 1 *μ*l of cDNA in a 20 *μ*l final reaction volume. The PCR thermal cycle conditions were as follows: initial step at 95°C for 10 min, followed by 40 cycles of 95°C for 15 s and 60°C for 1 min. The expression level of the housekeeping gene *β*_2_-microglobulin was measured as an internal reference with a standard curve to determine the integrity of template RNA for all specimens. The ratio of the mRNA level of each gene was calculated as follows: (absolute copy number of each gene)/(absolute copy number of *β*_2_-microglobulin).

### Animal and cell line

Female C57BL/6 mice (8–12-weeks old) were obtained from CLEA JAPAN (Tokyo, Japan). All mice were maintained under specific pathogen-free conditions in the animal facility at Nara Medical University. All experiments were conducted under a protocol approved by our institutional review board. A murine pancreatic adenocarcinoma, PAN02, was obtained from the DCTD Tumor Repository, National Cancer Institute (Frederick, MD, USA). Cells were grown in RPMI 1640 supplemented with 10% heat-inactivated foetal bovine serum.

### Animal experimental protocol

PAN02 was orthotopically implanted in the pancreas of syngeneic C57BL/6 mice. The anti-mouse B7-H3-blocking monoclonal Ab (MJ18, rat IgG1) was generated as previously described (Nagashima *et al*, 2008). The day after tumour implantation, mAb treatment was started. In the antibody treatment arm, mice were intraperitoneally injected with 0.3 mg of MJ18 every other day for 3 weeks. Control mice received control rat IgG. For *in vivo* depletion of CD8^+^ T cells, mice received an anti-CD8-depleting monoclonal Ab (2.43, rat IgG, 200 *μ*g), which was intraperitoneally injected 6, 3, and 1 day before tumour implantation and weekly thereafter as previously described (Sho *et al*, 2002). Gemcitabine was intraperitoneally given at a dose of 0.8 mg per body, every 3 days, 5 times in total. The doses were determined on the basis of our preliminary experiments. Tumour volume was calculated according to the formula V=A × B^2^/2 (mm^3^), where A is the largest diameter (mm) and B is the smallest diameter (mm). At 4 weeks after tumour establishment, the mice were killed and tumours were removed for further analysis.

### Cell viability analysis

Cell viability was determined using the Cell-titer 96 aqueous one solution cell proliferation assay kit, according to the instruction manual (Promega Corporation, Madison, WI, USA). Briefly, aliquots of 1 × 10^3^ cells per well were cultured in 96-well plates with MJ18 or control IgG for 72 h. Antibody was used at a concentration of 1 or 10 *μ*g ml^−1^. Cell-titer 96 aqueous one solution was added to each well and incubated for an additional 1 h. The absorbance at 490 nm was recorded with a 96-well plate reader. Each experiment was performed in triplicate and repeated at least thrice.

### Statistical analysis

Results were expressed as mean values±standard error, and Student's *t*-test was used for evaluating statistical significance. A value less than 0.05 (*P*<0.05) was considered for statistical significance.

## Results

### B7-H3 expression in human pancreatic cancer

**Figure 1:**
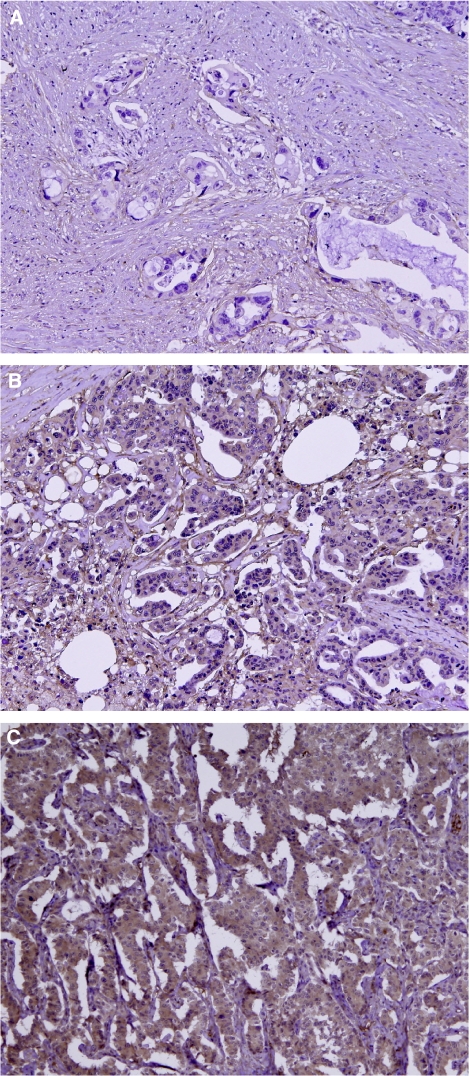
Immunohistochemical staining of human pancreatic cancer tissue for B7-H3. Positive staining was seen on the cell membrane and in the cytoplasm of cancer cells in almost all patients. Representative tissue of no staining (**A**), weak intensity (**B**), and strong intensity (**C**) for B7-H3 expression in pancreatic cancer was shown. Original magnification, × 100.

**Figure 2:**
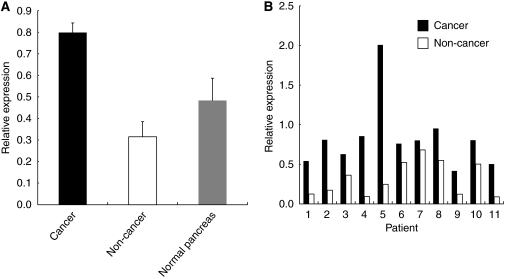
Comparison of B7-H3 expression between cancer and non-cancer tissues of the pancreas. (**A**) The cumulative B7-H3 expression in cancer tissue (*n*=30) was significantly higher compared with that in non-cancer tissue (*n*=11) and normal pancreatic tissue (*n*=5) (cancer *vs* non-cancer, *P*<0.0001; cancer *vs* normal pancreas, *P*=0.0078; non-cancer *vs* normal pancreas, N.S.). (**B**) The expression in cancer tissue is consistently higher than that in non-cancer tissue of individual pancreatic cancer patients.

First, we examined the expression of B7-H3 on 59 pancreatic cancer tissues by immunohistochemistry. Positive staining was seen both on the cell membrane and in the cytoplasm of cancer cells in 55 patients (93.2%), whereas only 4 patients (6.8%) had no staining for B7-H3 (Figure 1). In non-cancer tissues, some islet cells were also positive for B7-H3. We then compared the relative expression of B7-H3 between pancreatic cancer and non-cancer tissues using available frozen tissues by quantitative real-time PCR. The B7-H3 expression in cancerous pancreatic tissues was significantly higher than in non-cancerous or normal pancreatic tissues (cancer *vs* non-cancer, *P*<0.001; cancer *vs* normal pancreas, *P*=0.008; non-cancer *vs* normal pancreas, N.S.) (Figure 2A). Furthermore, the B7-H3 expression of cancer tissue was consistently higher than that of non-cancer tissue in each individual pancreatic cancer patient (Figure 2B). These data suggested that B7-H3 might have some effect and may be a potential target for immunotherapy in pancreatic cancer.

### Correlation between B7-H3 expression and pathological findings

B7-H3 was positively stained in over 90% of pancreatic cancer patients. In these 55 positive tissues, it was homogeneously expressed in almost all pancreatic cancer cells in each examined tumour section. Therefore, all specimens were classified into two groups according to staining intensity as follows: 39 tumours with strong staining and 20 tumours with weak or no staining (Figure 1). We evaluated the correlation of the B7-H3 expression with various clinicopathological data. We found that tumours with a strong intensity of B7-H3 expression had more common lymph node metastasis (*P*=0.020) and advanced pathological stage (*P*=0.040) (Table 1). These data suggested that B7-H3 expression might be functionally important in tumour progression and metastasis in pancreatic cancer. In this study, there was no significant difference in the post-operative prognosis between strong and weak intensity of tumour B7-H3 expression.

### Therapeutic efficacy of anti-B7-H3 mAb in pancreatic cancer

**Figure 3:**
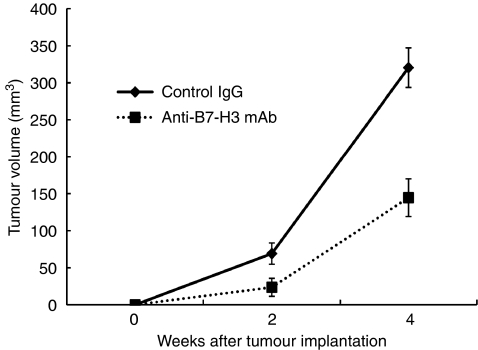
Therapeutic efficacy of B7-H3 blockade in murine pancreatic cancer. The effect of B7-H3 blockade on pancreatic cancer was examined *in vivo*. PAN02 was orthotopically implanted in the pancreas of syngeneic C57BL/6 mice. B7-H3 blockade significantly inhibited tumour growth compared with controls. (*P*=0.036 and 0.0002 at 2 and 4 weeks, respectively).

**Figure 4:**
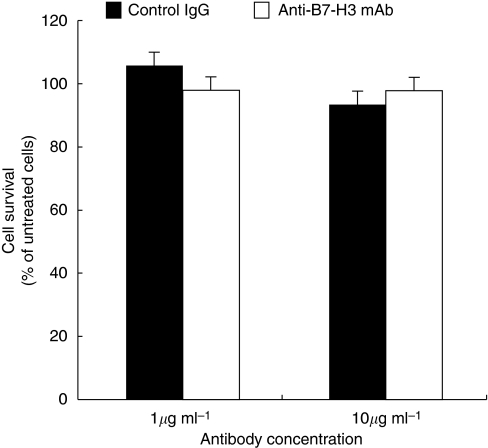
Effect of anti-B7-H3 mAb on pancreatic cancer *in vitro*. A total of 1000 murine pancreatic cancer cells, PAN02s, were co-cultured with anti-B7-H3-blocking mAb, MJ18, or control IgG. B7-H3 blockade did not have any direct effect on cancer cell growth *in vitro,* as determined by MTS assay.

**Figure 5:**
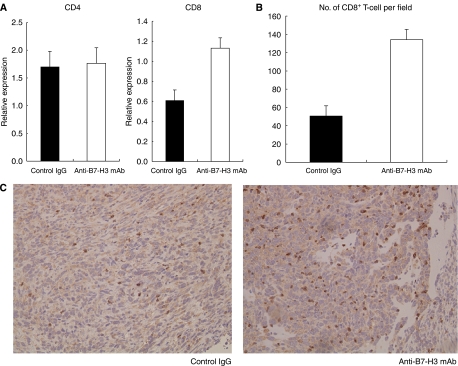
Tumour-infiltrating T cells. (**A**) At 2 weeks after tumour implantation, CD8^+^, but not CD4^+^, T cells in tumours treated with anti-B7-H3 mAb were more abundant than in controls (*P*=0.024), as shown by quantitative real-time PCR. (**B**) Immunohistochemical analysis of CD8^+^ T cells in tumours treated with anti-B7-H3 mAb or control IgG (*P*<0.001). (**C**) Representative immunohistochemical staining of tumour-infiltrating CD8^+^ T cells. Original magnification, × 200. A more profound infiltration of CD8^+^ T cells was observed in the tumour treated with anti-B7-H3 mAb compared with controls.

**Figure 6:**
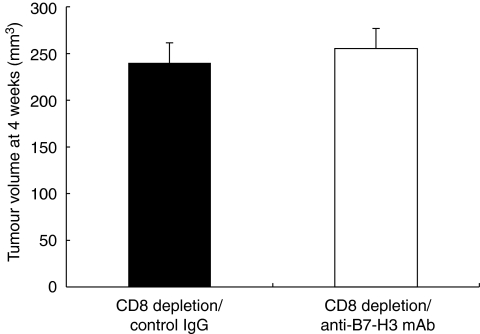
Requirement of CD8^+^ T cells for therapeutic efficacy of B7-H3 blockade. The tumour size between the control group and the treatment group did not have any statistical difference at 4 weeks after tumour implantation in mice depleted of CD8^+^ T cells.

Towards a clinical application of targeting the B7-H3 pathway, we examined the therapeutic efficacy of anti-B7-H3-blocking mAb in pancreatic cancer. To this end, we used a murine pancreatic cancer model using PAN02, a murine pancreatic adenocarcinoma. We confirmed B7-H3 expression in PAN02 by RT-PCR and flow cytometry (data not shown). The anti-B7-H3 mAb, MJ18, treatment induced substantial anti-tumour effect *in vivo* and significantly inhibited tumour growth (tumour volume at 2 weeks: anti-B7-H3 mAb, *n*=7, 23.6±12.2 mm^3^; control, *n*=5, 69.1±14.4 mm^3^; *P*=0.036; tumour volume at 4 weeks: anti-B7-H3 mAb, *n*=10, 144.6±13.3 mm^3^; control, *n*=9, 320.4±36.1 mm^3^; *P*=0.0002) (Figure 3). To analyse the underlying mechanisms, we first tested *in vitro* effect of MJ18 on PAN02. A total of 1000 PAN02s were co-cultured with MJ18. Control rat IgG was used as a control. The survival rate of PAN02 was determined by MTS assay. As a result, B7-H3 blockade did not have any direct effect on cancer cell growth *in vitro* (Figure 4). We then evaluated tumour-infiltrating T cells after mAb treatment. At 2 weeks after tumour implantation, CD8^+^, but not CD4^+^, T cells in tumours treated with MJ18 were significantly more than that in controls, as indicated by real-time PCR (CD8^+^ T cells, *n*=3, *P*=0.024; CD4^+^ T cells, *n*=3, N.S.; controls, *n*=4) (Figure 5A). This was confirmed by immunohistochemical analysis on pancreatic cancer tissues at 4 weeks after tumour implantation. There was a more profound infiltration of CD8^+^ T cells into the tumour treated with B7-H3 blockade compared with controls (anti-B7-H3 mAb, *n*=5, 134.2±11.2; control, *n*=5, 50.5±11.2, *P*<0.001) (Figure 5B and C). Data indicated that B7-H3 blockade promoted the infiltrations of CD8^+^ T cells into tumours, thereby resulting in tumour growth inhibition. Furthermore, to confirm the requirement of CD8^+^ T cells for the anti-tumour effect of B7-H3 blockade, we used an *in vivo* depletion experiment. As a result, the depletion of CD8^+^ T cells completely abolished the effect of anti-B7-H3 mAb on murine pancreatic cancer (tumour volume at 4 weeks: anti-B7-H3 mAb, *n*=5, 255.4±21.4 mm^3^; control, *n*=5, 240.1±21.4 mm^3^; N.S.) (Figure 6). Therefore, data clearly indicated that CD8 T cells are effectors and are also essential for the anti-tumour effect of B7-H3 blockade.

### Synergy between B7-H3 blockade and conventional chemotherapy

**Figure 7:**
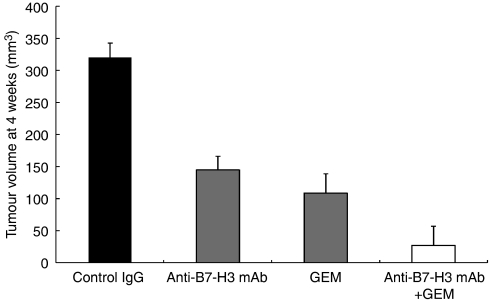
Combination of B7-H3 blockade with gemcitabine. Treatment with gemcitabine (GEM) alone resulted in a significant inhibition of tumour growth (*P*=0.0011). Furthermore, the combined treatment with gemcitabine and B7-H3 blockade showed a substantial synergistic anti-tumour effect (*P*<0.0001 compared with gemcitabine alone or B7-H3 blockade alone).

Finally, we evaluated the combination of conventional chemotherapy with B7-H3 blockade in pancreatic cancer. We used gemcitabine, which is currently the standard chemotherapeutic agent for pancreatic cancer. The treatment with gemcitabine alone resulted in significant inhibition of tumour growth (*n*=5, 108.6±9.3 mm^3^, *P*=0.0011 compared with controls). The combined treatment of gemcitabine and B7-H3 blockade showed a substantial synergistic anti-tumour effect on pancreatic cancer (*n*=5, 27.1±4.9 mm^3^, *P*<0.0001 compared with gemcitabine alone or B7-H3 blockade alone) (Figure 7). There were no overt toxicity and death in mice during and after treatment.

## Discussion

Although the cancer-related death rates of various malignancies have been declining because of improvements in early detection and treatment, the overall pancreatic cancer mortality rate is still extremely high (Jemal *et al*, 2007). To improve patient survival, novel strategies need to be developed. Immunotherapy is an attractive strategy and may provide a breakthrough in the treatment of pancreatic cancer. However, the clinical response rates of current immunotherapies are limited, presumably because of tumour immune escape mechanisms (Laheru and Jaffee, 2005; Plate, 2007). Thus, to elicit sufficient tumour-specific T-cell responses, it is necessary to overcome tumour immune evasion. One of the strategies may be targeting T-cell-inhibitory pathways, including B7/CTLA-4 and PD-L/PD-1 (Dong *et al*, 2002; Curiel *et al*, 2003; Ohigashi *et al*, 2005; Nomi *et al*, 2007). However, the effect of human CTLA-4-blocking antibody is likely to be limited in current clinical trials (Downey *et al*, 2007; Hodi *et al*, 2008). Therefore, further attempts to identify specific negative regulatory molecules for cancer immunotherapy may be needed. We were intrigued with a new member of the B7 family, B7-H3, as a potential negative molecule for T-cell activation in tumour immunity. We first examined the tumour B7-H3 expression in human pancreatic cancer, and found that tumour B7-H3 expression was consistently high in comparison with non-cancer tissue and significantly correlated with lymph node metastasis and advanced pathological stage. These clinical data suggested that tumour B7-H3 might function as a negative regulator in pancreatic cancer. This might be consistent with several previous reports in other malignancies, including lung and prostate cancers (Castriconi *et al*, 2004; Sun *et al*, 2006; Roth *et al*, 2007; Zang *et al*, 2007). However, there was no significant difference in post-operative prognosis as a result of tumour B7-H3 expression. The reason for this discrepancy may lie, in part, in the development of adjuvant chemotherapy. In fact, the post-operative prognosis in our department is much better because of aggressive chemotherapy for post-operative recurrence. On the other hand, conflicting observations were shown in a clinical study of gastric cancer and in a study of a murine colon cancer model. These indicate that high intra-tumour B7-H3 expression correlated with better prognosis in gastric cancer (Wu *et al*, 2006), and the introduction of B7-H3 into colon cancer cells leads to a reduction in tumour metastasis and tumour regression (Lupu *et al*, 2006, 2007). To clarify these discrepancies in tumour B7-H3 expression, a key issue may be to identify unknown receptors for B7-H3. It has been reported that soluble B7-H3 protein binds a putative counter-receptor on activated T cells that is distinct from CD28 and CTLA-4 (Chapoval *et al*, 2001; Sun *et al*, 2002). Furthermore, two distinct receptors have been assumed to explain the duality of the stimulatory and inhibitory functions imparted by B7-H3 (Roth *et al*, 2007). Hashiguchi *et al*, 2008 have recently reported that the triggering receptor expressed on myeloid cells, (TREM)-like Transcript 2 (TLT-2, TREML2), is a receptor for B7-H3. They showed that the interaction of B7-H3 with TLT-2 on T cells enhanced T-cell activation. B7-H3 may use another inhibitory receptor besides TLT-2. Therefore, depending on the affinity of differential receptors, tumour-associated B7-H3 may have distinct functional effects *in vivo*. A high-level expression of B7-H3 by transfection may engage TLT-2 to enhance T-cell responses. By contrast, other cancers with relatively low or physiological levels of B7-H3 expression may engage an inhibitory receptor (Lupu *et al*, 2006, 2007; Yi and Chen, 2009). Further investigations for the identification of receptors are clearly warranted to elucidate the physiological and pathological functions of B7-H3 in various conditions.

On the basis of our clinical data, we then investigated the therapeutic efficacy of blocking the B7-H3 pathway in pancreatic cancer towards future application. We used a murine orthotopic pancreatic cancer model. We found several important observations. First, anti-B7-H3-blocking mAb had a significant anti-tumour effect on tumour growth *in vivo*. This anti-tumour effect was not observed *in vitro*, suggesting that mAb had no direct effect on cancer cell growth. Furthermore, we observed that CD8^+^ T-cell infiltration into tumours was promoted by B7-H3 blockade. Furthermore, the *in vivo* depletion indicated that CD8^+^ T cells are required for the anti-tumour effect of B7-H3 blockade. Taken together, these data suggested that the B7-H3 pathway might critically regulate the growth of pancreatic cancer *in vivo* through the negative interaction between tumours and tumour-reactive CD8^+^ T cells. Although some islet cells were also positive for B7-H3, blood sugar levels were consistently normal and mice were healthy after mAb treatment. Second, more importantly, our data indicated that the combination of gemcitabine with B7-H3 blockade exerted a synergistic anti-tumour effect on pancreatic cancer. In clinical settings, gemcitabine is still currently the best treatment available for pancreatic cancer (Burris *et al*, 1997; Li *et al*, 2004; Hochster *et al*, 2006; Oettle *et al*, 2007). However, the effect of gemcitabine alone is limited and most patients develop resistance to the therapy. Therefore, gemcitabine, in combination with other approaches, is currently under investigation (Hochster *et al*, 2006). Chemotherapy and immunotherapy have usually been regarded as unrelated or potentially antagonistic forms of therapy (Lake and Robinson, 2005). This is because most chemotherapies kill target cells by apoptosis and similarly induce cell death of activated T cells recognising tumour antigen. In addition, lymphopenia is a common side effect of many anti-cancer drugs and this has been assumed to be detrimental to sufficient anti-tumour immune response. On the other hand, a number of studies have showed that chemotherapy could synergise with immunotherapy under some circumstances. In fact, it has been reported that gemcitabine reduces myeloid-derived suppressor cells, accompanied by an increase in the anti-tumour activity of CD8^+^ T cells and activated NK cells (Suzuki *et al*, 2005). In addition, several previous animal studies have shown a synergistic anti-tumour effect of gemcitabine with certain immunotherapies, including cytokines, monoclonal antibodies, and vaccines (Nomi *et al*, 2007). Thus, gemcitabine is a candidate chemotherapeutic agent for the combination therapy with immunotherapy. Although further studies are required to determine the underlying mechanisms in a synergistic effect between gemcitabine and B7-H3 blockade, our data may be clinically important and support future application of B7-H3 blockade for the treatment of pancreatic cancer.

In conclusion, we showed for the first time that B7-H3 is abundantly expressed in human pancreatic cancer and it has clinical significance. Furthermore, our data also indicated that B7-H3 is a potential target for immunotherapy against pancreatic cancer. This study may provide the rationale for developing a novel cancer therapy for this fatal malignant disease.

## Figures and Tables

**Table 1:** Clinicopathological characteristics according to tumor B7-H3 expression

		**B7-H3 intensity**		
**Variable**	***n***	**None or weak (%)**	**Strong (%)**	***P*-value**
*Tumour status*				
T1	3	1 (5.0)	2 (5.1)	0.646
T2	12	5 (25.0)	7 (18.0)	
T3	26	10 (50.0)	16 (41.0)	
T4	18	4 (20.0)	14 (35.9)	
				
*Nodal status*				
N0	26	13 (65.0)	13 (33.3)	0.020
N1	33	7 (35.0)	26 (66.7)	
				
*Metastatic status*				
M0	52	18 (90.0)	34 (87.2)	0.751
M1	7	2 (10.0)	5 (12.8)	
				
*Pathological stage*				
IA, IB	7	4 (20.0)	3 (7.7)	0.040
IIA	13	8 (40.0)	5 (12.8)	
IIB	16	4 (20.0)	12 (30.8)	
III	16	2 (10.0)	14 (35.9)	
IV	7	2 (10.0)	5 (12.8)	
